# Effects of resistance training and aerobic exercise in elderly people concerning physical fitness and ability: a prospective clinical trial

**DOI:** 10.1590/S1679-45082013000200003

**Published:** 2013

**Authors:** Maria Fernanda Bottino Roma, Alexandre Leopold Busse, Rosana Aparecida Betoni, Antonio Cesar de Melo, Juwando Kong, Jose Maria Santarem, Wilson Jacob

**Affiliations:** 1 Hospital das Clínicas, Faculdade de Medicina, Universidade de São Paulo, São Paulo, SP, Brazil

**Keywords:** Motor activity, Pliability, Postural balance, Muscular strength, Resistance training, Aged, Health of the elderly

## Abstract

**Objective:**

To compare the effects of physical fitness and function on older adults in two programs of supervised exercise activity: resistance training and aerobic exercise.

**Methods:**

This study is a randomized, prospective clinical trial composed of sedentary elderly people who did not have contraindications to exercise. Participants were divided into two groups: group one performed 6 exercises of resistance training twice a week, and group two participated in walking activity for 30 minutes twice a week. Functional assessment (time 0,6 and 12 months) was measured by the Short Physical Performance Battery (time to sit or stand, gait speed, and balance), flexibility test, and the six-minute walking test. We randomly selected 96 patients: 46 in the Resistance Training Group and 50 in the Aerobic Exercise Group. In the Resistance Training Group, 46 attended the first assessment and 20 attended until the third section. In the Aerobic Exercise Group, 50 attended the first assessment and 12 attended until the third assessment.

**Results:**

Mean age was 68.8 years in the Resistance Training Group and 69.1 years in the Aerobic Exercise Group. The Resistance Training Group showed improvement in the sit/ stand (p=0.022), balance with feet in a row (p=0.039) and queued (p=0.001). The second showed a statistical difference in speed and balance with the feet lined up and the feet together (p=0.008; p=0.02; and p=0.043, respectively). Concerning flexibility, the Resistance Training Group had improvement (p=0.001), whereas in the Aerobic Exercise Group, no significant difference was seen (p=0.359). Both groups had improvement in the six-minute walking test, but no significant improvement was seen in the Aerobic Exercise Group (p=0.033).

**Conclusion:**

Both groups showed improvement in physical fitness. No statistical difference was seen when groups was compared in the short physical performance battery, flexibility, and six-minute walking test.

Clinical trial register: UTN: U1111-1141-3066

## RESUMO

**Objetivo:**

Comparar os efeitos das atividades físicas resistida e aeróbia sobre a aptidão física e funcionalidade de idosos em dois programas de atividade supervisionada: exercícios resistidos e caminhada.

**Métodos:**

Ensaio clínico, randomizado, prospectivo, com idosos sedentários, sem contraindicações para atividade física, distribuídos em dois grupos: o Grupo Resistido realizou 6 exercícios por treino, 2 vezes por semana e o Grupo Aeróbio realizou atividade por 30 minutos, 2 vezes por semana. A avaliação funcional (tempo 0,6 e 12 meses) foi realizada pelos seguintes parâmetros: *Short Physical Performance Battery* (tempo de sentar/levantar, velocidade da marcha e equilíbrio), flexibilidade, teste de caminhada de 6 minutos. Foram randomizados 96 participantes, 46 no Grupo Resistido e 50 no Grupo Aeróbio. No resistido, 46 compareceram na primeira avaliação e 20 permaneceram até a terceira. No aeróbio, 50 compareceram na primeira avaliação e 12 permaneceram até a terceira.

**Resultados:**

A média da idade dos pacientes no Grupo Resistido foi de 68,8 anos e de 69,1 no Grupo Aeróbio. O primeiro apresentou melhora no sentar/ levantar (p=0,022), no equilíbrio com pés seguidos (p=0,039) e enfileirados (p=0,001). No segundo, houve diferença estatística na velocidade, equilíbrio com pés seguidos e enfileirados (p=0,008, p=0,02 e p=0,043, respectivamente). Quanto à flexibilidade, o Grupo Resistido apresentou melhora (p=0,001), enquanto no Aeróbio não houve diferença significativa (p=0,359). No teste de caminhada de 6 minutos, ambos melhoraram, mas apenas o Grupo Aeróbio com significância (p=0,033).

**Conclusão:**

Ambos os grupos apresentaram melhora na aptidão física. Não houve diferença estatística quando comparados os grupos em relação ao *Short Physical Performance Battery,* à flexibilidade e à caminhada de 6 minutos na amostra estudada.

Registro do ensaio clínico: UTN: U1111-1141-3066

## INTRODUCTION

Functionality could be described as personal competency to perform daily life activities in a safe and independent way and without fatigue^(1,2)^. It is directly associated with strength and muscular potency as stated by Bassey et al. and Skelton et al.^(1,2)^, and Skelton et al, flexibility, aerobic capability, agility, and balance. Functional assessment could be performed using simple tests such as elevate a chair, static balance, and gait speed. The Short Physical Performance Battery (SPPB), standardized by Guralnik in 1995^(3)^, includes these three tests that can be performed rapidly and easily; therefore, it is widely used both in clinical practice and clinical studies.

The aging process causes a quantitative loss of muscle mass (sarcopenia) and a decrease in strength and in muscle potency. The peak of muscle strength occurs between the second and third decade of life. Up to age 50 years, a slight decrease in muscle strength occurs, which is stressed after age 65 years, and then decreases 12% to 15% for each decade^(3,4)^. There is also a qualitative reduction in muscle strength with atrophy of the fast fibers (type II fibers), reduction in tendon elasticity, and low activation of agonist and higher antagonist muscles^(5)^.

Loss of muscle occurs at different levels in ranges or in muscle groups. The proximal muscles of the lower extremities are more affected than the upper extremities^(6-8)^.

The benefits of resistance training – increased muscle strength and muscle mass – are well defined in the literature^(1,4)^, and aerobic activity has been known to help prevent cardiovascular disease.

## OBJECTIVE

To compare effects of physical fitness (muscle strength, balance, and flexibility) on functionality in older adults in two programs of supervised exercise activity that included resistance and aerobic activities.

## METHODS

Our study, a randomized clinical trial, compares two groups (resistance training and aerobic activity) who participated in physical activity for 12 months (January to December 2009). The study population was composed of elderly people, citizens of São Paulo who were recruited by advertisements from local newspapers, radio, and the Internet.

We included participants older than 60 years who lived in the city of São Paulo, did not have contraindications to exercise, and who were sedentary (have not participated in regular exercise in the last 6 months). Those who were excluded had non-compensated diabetes mellitus (fasting glycemia >250mg/dL), severe arrhythmia, acute myocardial infarction for at least 6 months, aortic aneurysm, severe aortic stenosis, angina of effort (for 2 months), and uncontrolled systemic arterial hypertension (systolic blood pressure >180mmHg and diastolic blood pressure >110mmHg).

**Figure 1:**
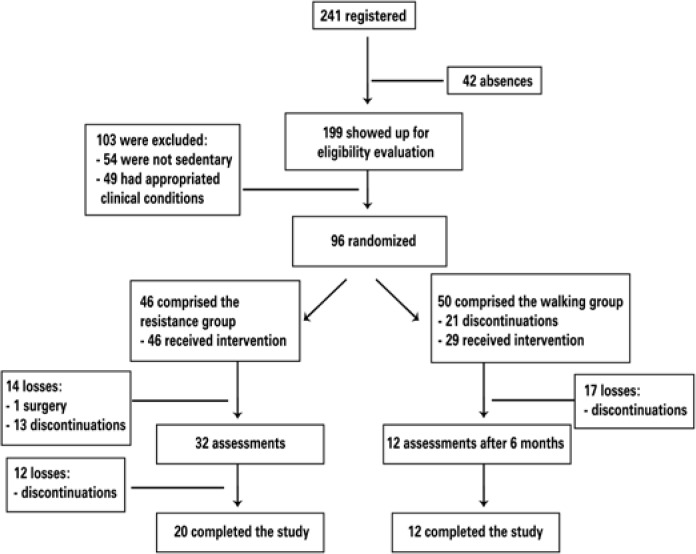
Flowchart

Of 241 volunteers, 96 were included, and participants were randomly divided into two groups: a Resistance Group (RG) and an Aerobic Group (AG) (Figure 1). The random process was done using drawn done with pieces of paper inside a plastic bag (50 vacancies for muscle building and 50 vacancies for walking). All participants signed a consent form before the program began. This study was approved by the Ethical and Research Committee of the *Hospital das Clínicas e da Faculdade de Medicina* from the *Universidade de São Paulo* (FMUSP), protocol number 0614/09.

### Assessment

Functional assessment was conducted at time 0,6 and 12 months.

Using the SPPB (Guralnik test), scores ranged from 0 to 12 points:

– Sit and stand up (S/S): time to perform five repetitions to sit and rise from a chair without using one's arms. Time ≤11.1 seconds was counted as 4 points; between 11.2 and 13.6 seconds, 3 points; between 13.7 and 16.6 seconds, 2 points; and ≥16.7 seconds, 1 point. If the task was not accomplished, no points were given.
– Gait speed: time spent to cover 2.4 meters. Time ≤3.1 seconds was counted as 4 points; between 3.2 and 4.0 seconds, 3 points; between 4.1 and 5.6 seconds, 2 points; and ≥5.7 seconds, 1 point. If the task was not accomplished, no points were given.
– Balance: with parallel feet (PF), feet together (FT) and lined up feet (LF). Individuals were required to stay for 10 seconds in each position. Those who stayed for 10 seconds in each position requested had 4 points. A total of 3 points were given for those who stayed for 10 seconds in the PF position and in the FT position, and from 3 to 9 seconds in the LF position; 2 points were given for those who stayed for 10 seconds in the PF position and in the FT position, and until 2 seconds in the LF position; and 1 point was given for those who stayed for 10 seconds in the PF position and less than 10 seconds in the FT position. No points were given for those who stayed for less than 10 seconds in the LF position.

Flexibility test (Wells bench): the patient was seated with back straight, knees straight, and feet rested on a box. The point where the hands reached was measured in centimeters (farthest distancing point reached with hands, keeping the knees straight).

In the six-minute walking test, the distance (in meters) reached was measured during the test, and participants needed to walk as fast as possible.

### Resistance activity program

Resistance exercises were performed at an experimental therapeutic gymnasium. The RG participated in the exercises for 12 months, and training lasted for 1 hour twice a week. Exercises were done using six types of equipments designed for elderly people from Maxiflex line (Biodelta^®^), of lifting and weighting system, without wires or pulleys. In each exercise, loads were progressively increased in sets of 12, 10, and 8 repetitions. Exercises included chest presses, rows, leg presses, calf presses, sit-ups, and lower back exercises.

### Aerobic activity program

Aerobic activity was done on a walking track. The AG did this activity for 12 months, and training lasted for 30 minutes twice a week. Heart rate (HR) was measured every 5 minutes with, the aim of keeping it between 60% and 70% of maximal HR (220 – age). The participant was encouraged to increase intensity if the HR was less than expected, or to decrease the intensity if it was more than what was expected.

### Statistical analysis

For data analysis, we used the Statistical Package for the Social Science (SPSS) V16 and Minitab 15.

Tests and nonparametric techniques were used. The Friedman test was used to compare three assessments, and the Wilcoxon test was used to compare by pairs.

The significance adapted to all analyses was p<0.05. Values were expressed in mean ± standard deviation.

## RESULTS

The study population was composed of mostly women (85.4%) between 60 and 86 years old (68 ± 5.9). Groups were similar in relationship to beginner features (Table 1).

**Table 1:** Initial characteristics

Characteristis	Group (n)		p value
	RG (46)	AG (50)	
Women	40 (86.9)*	42 (84)*	0.770
Age (years)	68.8 (5.6)	69.1 (5.6)	0.988
Height (meters)	1.58 (0.008)	1.59 (0.009)	0.820
Weight (kg)	71 (14.5)	72.4 (16.2)	0.752
BMI (kg/cm^2^)	28.2 (4.1)	28.5 (5.1)	0.866
SBP (mmHg)	130.7 (21.5)	135.7 (17.1)	0.225
DBP (mmHg)	80.3 (11.6)	83.1 (12.5)	0.419

Values expressed in means (standard deviation).

* RG: resistance group, AG: aerobic group, BMI: body mass index; SBP: systolic blood pressure; DBP: diastolic blood pressure.

In the RG of 46 participants, 20 (43.5%) completed 12 months of the study. One participant discontinued the program because of surgery that was unrelated to the exercise practice; the remaining participants dropped out of the study for personal reasons.

In the AG of 50 participants, 12 (24%) completed 12 months of the study. All who discontinued the study did so for personal reasons.

In the flexibility test, the RG showed a significant improvement (22.1 *versus* 24.0 seconds; p=0.001), whereas no statistical improvement was seen in the AG (22.5 *versus* 23.4 seconds; p=0.359). In the six-minute walking test, the RG showed a significant improvement (508.6 *versus* 530.5 seconds; p=0.538), which was different from the AG (500.6 *versus* 548.4 seconds; p=0.033) (Table 2 and 3).

**Table 2:** Results of walking test lasting for 6 minutes

Groups		First assessment	Second assessment	Third assessment	p value
Resistance	n	46	32	20	
	MD/SD	508.6 (±70)	535.8 (±67.6)	530.5 (±54.8)	0.538
Walking	n	50	12	12	
	MD/SD	500.6 (±74)	571 (±85.6)	548.4 (±86.5)	0.033

MD: medium; SD: standard deviation.

**Table 3:** Results of flexibility test

Groups		First assessment	Second assessment	Third assessment	p value
Resistance	n	46	32	20	
	MD/SD	22.1 (±7.2)	24.9 (±7.3)	24 (±6.8)	0.001
Aerobic	n	50	12	12	
	MD/SD	22.7 (±7.2)	26.4 (±9.7)	23.4 (±8.1)	0.359

MD: medium; SD: standard deviation.

In the SPPB, the RG showed improved outcomes in S/S 8.8 *versus* 6.8 seconds; p=0.022), in the balance with FT (8.2 *versus* 10.0 seconds; p=0.039) and with LF (6.6 *versus* 10.0 seconds; p=0.001). The AG had improvement in speed (1.5 *versus* 1.38 seconds; p=0.008), in balance with FT (7.6 *versus* 10.0 seconds; p=0.021), and with LF (6.2 *versus* 9.8 seconds; p=0.043). When comparing the total SPPB score (S/S + balance + speed) with a minimal score of 0 and maximal score of 12, we found a statistical difference in RG (p=0.005) and in AG (p=0.014) (Table 4).

**Table 4:** Total score of Short Physical Performance Battery

Groups		First assessment (%)	Second assessment (%)	Third assessment (%)	p value
Resistance	7 a 9*	30.40	18.80	0	0.005
	10 a 12*	69.60	81.30	100	
Aerobic	7 a 9*	36	33.30	0	0.014
	10 a 12*	64	66.70	100	

* Total score of Short Physical Performance Battery.

## DISCUSSION

Findings in our study reinforced the value of physical activity for elderly people to improve physical fitness and, as a result, functionality. Our results showed that both resistance and aerobic activity have a positive effect on functioning maintenance.

Guralnik et al.^(3)^ have concluded that scores between 4 and 6 points are 4.2 to 4.9 times, respectively, more related to a decrease in functionality at 4 years compared with scores between 10 and 12 points. Compared with scores between 7 and 9 points, scores between 10 and 12 points are 1.6 to 1.8 times, respectively, more related to a decrease in functionality. In our study, after 12 months all participants scored between 10 and 12 points on the Guralnik test, which highlighted in both groups a lower risk for functioning loss during 4 years.

One problem observed in our study was that the volunteers discontinued participation in the program at 12 months. In the RG, adherence was 43%; in the AG, it was 24%. Adherence to the physical activity program lasting for more than nine months is a well-known challenge in the literature. A meta-analysis conducted in 2008 concluded that adherence to a program lasting for 12 months ranged from 42% to 100%^(9)^. One reason that could explain the participants' discontinuation in our study might be because the city of São Paulo is a metropolis with roughly 19 million people but has a deficient public transportation system, which makes it difficult for people to reach places easily. In addition, compared with other studies, adherence was lower in our study probably because of the different method of recruitment we used; other studies have recruited participants who were already followed up in hospitals^(10,11)^.

Our study showed a statistically significant improvement in walking for six minutes in the AG. The six-minute walking test is widely used to evaluate pulmonary rehabilitation and is considered reliable in the evaluation of functional capability^(12,13)^. Few studies have evaluated such a test in healthy individuals^(14,15)^. A prospective study of 4 years of follow-up with healthy elderly people with preserved functioning^(16)^ showed that worse performance in the lower limbs was a predictor of the development of functional loss. Another study showed a relationship between walking speed and muscle strength in the lower limbs^(16)^. Several studies have shown that hip strength is related to walking speed and improvement in functionality^(17-19)^. This finding indicates that aerobic activity is linked with improvement in functional capability.

Also a significant statistically improvement in flexibility was seen in GR. Flexibility represents an essential component to functional maintenance. A decrease in flexibility is associated with high injury of joints, bones, and muscles, and also with loss of functional capability^(20)^. Many studies have highlighted benefits of resistance activity in flexibility because of the use of broader movements to be performed ^(21)^.

A Cochrane review conducted in 2009 with 121 random clinical essays (6.700 participants) showed that elderly people who participated in resistance activity gained muscle strength. The same study also observed an improvement in execution of daily activities such as walking; climbing a ladder; elevate chairs; and also more complex tasks, such as taking a shower and cooking. This gain was high compared with gait speed^(22)^.

Guidelines and recommendations of physical activity for elderly people by the American Heart Association (AHA) and the American College of Sports Medicine (ACSM) in 2007^(23)^ highlight the importance of performing aerobic activity of moderate intensity for 30 minutes per day for at least 5 times a week, performing resistance activity 2 times a week on alternate days, and adding flexibility training for 10 minutes at least twice a week^(23,24)^.

Results of our study also suggested benefits of combined physical activity because whereas resistance activity improved flexibility, aerobic activity improved aerobic capability. Therefore, both components are fundamental to functional maintenance in the elderly population, as described above.

A limitation of this study was the discontinuation of patients during follow-up. Further studies involving more participants could show better improvement in both activities and also indicate benefits related to functionality of one type of activity in relationship to another.

## CONCLUSION

Both resistance and aerobic activity are efficient to improve physical fitness and functionality in the elderly population. In our study sample, the RG improved flexibility and static balance in S/S from the chair, and total score of the SPPB. On the other hand, AG improved gait speed, static balance, and total score of the SPPB.
